# The Effects of Folic Acid Administration on Cardiac Oxidative Stress and Cardiovascular Biomarkers in Diabetic Rats

**DOI:** 10.1155/2019/1342549

**Published:** 2019-06-11

**Authors:** Slavica Mutavdzin, Kristina Gopcevic, Sanja Stankovic, Jovana Jakovljevic Uzelac, Milica Labudovic Borovic, Dragan Djuric

**Affiliations:** ^1^Institute of Medical Physiology “Richard Burian”, Faculty of Medicine, University of Belgrade, Belgrade, Serbia; ^2^Institute of Chemistry in Medicine “Prof. Dr. Petar Matavulj”, Faculty of Medicine, University of Belgrade, Belgrade, Serbia; ^3^Centre of Medical Biochemistry, Clinical Centre of Serbia, Belgrade, Serbia; ^4^Institute of Histology and Embryology “Aleksandar Dj. Kostic”, Faculty of Medicine, University of Belgrade, Belgrade, Serbia

## Abstract

The aim of this study was to examine the effects of folic acid administration on the antioxidant enzyme (superoxide dismutase (SOD) and catalase (CAT)) activities, lactate and malate dehydrogenase (LDH and MDH) activities, and certain LDH and MDH isoform distribution in the cardiac tissue of diabetic Wistar male rats. Diabetes mellitus (DM) was induced by streptozotocin (STZ). There were five groups: C1—control (physiological saline 1 ml/kg, i.p. one day), C2—control with daily physiological saline treatment (1 ml/kg, i.p. 28 days), DM—diabetes mellitus (STZ 100 mg/kg in physiological saline, i.p. one day), FA—folic acid (5 mg/kg in physiological saline, i.p. 28 days), and DM+FA—diabetes mellitus and folic acid group (STZ 100 mg/kg in physiological saline, i.p. one day, and folic acid 5 mg/kg in physiological saline, i.p. 28 days). After four weeks, animal hearts were isolated for measurement of enzyme activities, as well as for histomorphometry analyses. An elevated glucose level and a decreased insulin level were obtained in the DM group. SOD, CAT, and MDH activities were elevated in the DM group, while there was no difference in LDH activity among the groups. In all tested groups, four LDH and three MDH isoforms were detected in the heart tissue, but with differences in their relative activities among the groups. Left ventricular cardiomyocyte transversal diameters were significantly smaller in both diabetic groups. Folic acid treatment of diabetic rats induced a reduced glucose level and reduced CAT, SOD, and MDH activities and alleviated the decrease in cardiomyocyte diameters. In conclusion, increased activities of antioxidant enzymes and MDH may be the consequence of oxidative stress caused by DM. Administration of the folic acid has a protective effect since it leads to reduction in glycemia and activities of the certain examined enzymes in the rats with experimentally induced DM.

## 1. Introduction

Diabetes mellitus (DM) has been defined as a group of disorders characterized not only by hyperglycemia but also by altered insulin action or secretion, altered metabolism of proteins, carbohydrates, and lipids, and the increased risk of vascular complications. Insulin resistance and hyperglycemia lead to dyslipidemia that is a risk factor for vascular diseases such as atherosclerosis and coronary artery disease [1]. The changes in the metabolism provoke damages of different tissues and organs [2]. Mechanisms of changes in the metabolism and energy synthesis are not completely clear, but it is known that they can lead to diabetic cardiomyopathy and decline in cardiac function [3]. Metabolism changes can be examined by lactate dehydrogenase (LDH) isoform detection and evaluation of these isoform activities. There are five isoforms of LDH (LDH1-LDH5). Their activities indicate the predominance of aerobic or anaerobic metabolism in a particular tissue, since it has been demonstrated that LDH1 isoform has increased activity in aerobic conditions, while LDH5 is more active in anaerobic conditions [4]. LDH catalyzes the oxidation of lactate to pyruvate when there are high concentrations of lactate and reverse reaction of the reduction of pyruvate to lactate occurs in case of oxygen deficiency in the cell [5]. LDH is a heterotetramer that consists of M and H subunits. In each of the LDH isoforms, there is a different ratio of the subunits which determines the affinity to lactate and pyruvate and the role of the isoform in the direct or reverse reactions [6]. LDH1 and LDH2 have higher affinity for lactate, and they are more active in the tissues well supplied with oxygen. LDH3 has the same affinity for both pyruvate and lactate. The last two isoforms dominate in the tissues where glycolysis predominates [7]. LDH, aspartate aminotransferase, and creatine kinase are cardiac marker enzymes which provide information about cardiomyocyte damage. The decrease in marker enzymes in the myocardium is a sign of the cellular injury due to lipid peroxides [8]. Assessment of these enzymes in the heart and/or in serum is important to estimate the cellular damage [8]. In addition to LDH, malate dehydrogenase (MDH) is an enzyme with important metabolic functions [9]. It catalyzes the conversion of malate into oxaloacetate, and *vice versa*. MDH has an important role in the citric acid cycle, and it is considered as a marker enzyme for this cycle [10, 11]. MDH is directly implicated in glucose metabolism [10, 12]. Mitochondrial MDH activity indicates overall metabolic status. The decrease of the cytosolic MDH reflects depression of energy metabolism in the cytoplasm [10, 13]. The MDH activity is a useful parameter to evaluate metabolic conditions both in the mitochondria and cytoplasm. Since DM leads to metabolic disorders, change in total MDH activity and its isoform distribution can also be expected.

Recently, it has been demonstrated that increased oxidative stress and changes in antioxidant capacity are an important mechanism in the pathogenesis of DM and its complications [14]. Oxidative stress may influence the endogenous antioxidant system that includes glutathione peroxidase (GSH-Px), superoxide dismutase (SOD), and catalase (CAT) antioxidant enzymes [15]. Prolonged hyperglycemia is followed by the production of reducing sugars. These sugars can react with lipids and proteins and increase the production of reactive oxygen species (ROS), which, consequently, increase oxidative stress. [16, 17]. Also, an elevated glucose level increases the production of reactive nitrogen species, such as peroxynitrite. It causes protein and DNA damage, activates polyADP ribose polymerase, and promotes endothelial stress and cell death [18]. It has been demonstrated that the oxidative stress alters protein, lipid, and carbohydrate metabolism [19] and may induce dysfunction of endothelial cells and cause the development of atherosclerosis. For that reason, it is considered to be the major player in the development of diabetic complications such as vascular, neuronal, ophthalmological, and renal disorders [17]. In order to prevent the damage by enhanced oxidative stress, the balance of ROS and antioxidants is necessary; hence, different supplements of antioxidants may be used to prevent consequences of oxidative stress [20].

Folic acid has an important role in reducing oxidative stress, improving endothelial function, and preventing apoptosis by reducing the plasma homocysteine level [21, 22]. In patients with DM and hyperhomocysteinemia, it has been demonstrated that folic acid lowers the plasma homocysteine level and improves endothelial function [23, 24]. There are also effects of folic acid that are independent of homocysteine, such as protection against endothelial nitric oxide synthase uncoupling, conservation of tetrahydrobiopterin, and inhibition of superoxide formation [25–27]. Also, independently on the homocysteine level, folic acid leads to an increase in coronary vasodilatation and to a mild decrease in arterial blood pressure in patients with coronary artery disease [28]. In addition, the perfusion of the isolated heart with folic acid significantly increased the flow through the coronary arteries and reduced the production of the superoxide anion; however, it increases the lipid peroxidation index [29]. Administration of the folic acid in diabetic rats at doses of 0.4 mg/kg/day and 1.2 mg/kg/day demonstrated a dose-dependent decrease in apoptosis of cardiomyocytes. It was also observed an increased level of antiapoptotic protein Bcl-2 and a reduced level of proapoptotic proteins Bax and Fas [30]. Since folic acid reduces oxidative stress and prevents cardiomyocyte apoptosis [21], increased LDH as well as mitochondrial and cytosolic MDH activity in the cardiac tissue in rats treated with folic acid is expected.

Despite the published studies, the effect of folic acid on the antioxidant enzymes in the cardiovascular system of both the healthy rats and those with DM type I is not completely clear, and further researches are needed. Thus, the aim of this study was to examine the influence of folic acid administration on the antioxidant enzymes, as well as on LDH and MDH activities and their certain isoform distribution in the cardiac tissue of diabetic Wistar male rats; furthermore, the influence of folic acid on the cardiovascular biomarkers and histological structure of the heart was tested.

## 2. Materials and Methods

### 2.1. Experimental Animals

Male Wistar albino rats (*n* = 46) with a body weight of approximately 160 g at the start of the experimental period were used in the research. The rats were housed in pairs in transparent plexiglass cages with a wood chip floor. The ambient conditions were constant (temperature 21 ± 2°C; relative humidity 55 ± 5%; 12 h light-dark cycle with the light period beginning at 07:30 a.m.). Standard food and water were available *ad libitum*. The study has been approved by the Ethical Council for the Welfare of Experimental Animals, Ministry of Agriculture, Forestry and Water Management, Veterinary Directorate, Republic of Serbia (number: 323-07-01339/2017-05/04). All experimental procedures were done in accordance with the prescribed legislation (EU Directive for the Protection of Vertebrate Animals Used for Experimental and Other Scientific Purposes 86/609/EES) and the principles of ethics.

### 2.2. Experimental Protocol

The research was conducted during a four-week period. The animals were distributed in five groups. There were two control groups: one (C1, *n* = 8) that received one dose of physiological saline (1 ml/kg b.w., i.p.) and the second one (C2, *n* = 10) that received physiological saline treatment during 28 consecutive days (1 ml/kg b.w., i.p.). The group C1 was introduced to examine whether daily treatment with physiological saline that exists in the C2 group may affect the tested parameters. The third group was the group of experimental animals with induced DM (DM, *n* = 8) using one dose of streptozotocin (STZ, 100 mg/kg b.w. in physiological saline, i.p.). The fourth group, the folic acid group (FA, *n* = 10), received folic acid (5 mg/kg b.w. in physiological saline, i.p.) treatment during 28 consecutive days. The fifth group was the group of experimental animals with induced DM and folic acid treatment (DM+FA, *n* = 10). First, they were treated with STZ (100 mg/kg b.w. in physiological saline, i.p., in one dose), and on the fourth day after STZ treatment, they received folic acid during the next 28 consecutive days.

The body weight and blood glucose level were measured at the start, weekly, and at the end of the experimental period. After four weeks of the treatment, the animals were euthanized using a rat guillotine. Blood was collected through a glass funnel and placed in appropriate vacutainers. The samples were left at the room temperature for 15 min and afterwards centrifuged (15 min × 3000 rpm). The obtained plasma and serum were used for the analyses. Prior to blood sample collection, animals were fasted overnight (during 12 h) to avoid food-induced changes in the blood biochemical parameters.

Whole hearts were isolated from all the rats. The hearts were rinsed in the physiological saline, and their weight was measured. After this procedure, the hearts from one half of the animals (randomly chosen) from each group were placed in formalin solution for histology analyses and the remaining hearts were homogenized and centrifuged for enzyme activity measurement.

### 2.3. Induction of Diabetes Mellitus in Rats

DM was induced in experimental animals by an intraperitoneal injection of freshly prepared STZ (100 mg/kg b.w.) [31], dissolved in physiological saline (0.9% NaCl, 1 ml/kg b.w.) [32–36]. Citrate buffer is used for dissolving STZ more frequently than physiological saline [2, 15, 17, 19, 30]. However, to avoid the effects of citrate buffer on the tested parameters, we used a physiological saline to dissolve STZ, but because of that, a higher dose of STZ is applied. An appropriate dose of STZ was previously determined following the dose-response relationship between different STZ doses (from 40 to 150 mg/kg) and the blood glucose level. 72 h after the STZ administration, DM was confirmed by measurement of the blood glucose level in animals that were fasting during previous 8 h. The values of glucose higher than 12.2 mmol/l were considered positive for DM [15].

### 2.4. Biochemical Parameter Determination

The glucose level in blood obtained from the rat tail vein in the animals that were fasting during previous 8 h was determined by an Accu-Chek analyzer (Roche Diagnostics, Indianapolis, USA). Serum homocysteine was measured by an automated electrochemiluminescence immunoassay system, ADVIA Centaur XP system (Siemens Healthcare GmbH, Erlangen, Germany). The serum glucose level, the lipid profile parameters (total cholesterol (TC), low-density lipoprotein (LDL), high-density lipoprotein (HDL), and triglycerides (TG)), and cardiac tissue damage parameters (LDH and troponin T) were analyzed using the spectrophotometer and commercial kits (Siemens Healthcare Diagnostics Ltd., Frimley, Camberley, UK) on an automatic biochemical analyzer (Dimension Xpand, Siemens). In order to confirm the development of DM, the serum insulin level was measured and homeostasis model assessment of insulin resistance (HOMA-IR) was calculated in the C1 and DM groups. The serum insulin level was determined by the RIA method, using rat insulin standards on an automatic counter (PerkinElmer GammaWIZARD 1470, Automatic Gamma Counter, Boston, USA). The rat fasting insulin reference level was 12.06–48.26 mU/l [37]. For the determination of haemostatic factors (fibrinogen and von Willebrand factor (vWF) activity), immunoturbidimetric commercial assay (Siemens Healthcare GmbH, Marburg, Germany) was used. Insulin resistance was evaluated by HOMA-IR using the formula: HOMA − IR = insulin (mU/l) × glucose (mmol/l)/22.5 [38]. Also, the atherogenic index (AI = LDL/HDL) as a marker of atherogenicity and increased ishaemic disease risk was calculated [39].

### 2.5. Heart Weight Index (HWI) Calculation

In order to evaluate the cardiac hypertrophy, the HWI was calculated using the formula: HWI = heart weight (mg)/body weight (g) [40].

### 2.6. Preparation of the Heart Tissue Samples

The heart tissue (100 mg) was homogenized on ice in 1 ml of buffer (20 mmol/l Tris-HCl, pH 7.5, 250 mmol/l sucrose, 1% Triton X-100, with the addition of the protease inhibitor: 1 mmol/l phenylmethylsulfonyl fluoride (PMSF) and 1 *μ*g/ml leupeptin) [41]. The homogenate was centrifuged at 10000 rpm at 4°C during 10 min. The total protein concentration was measured in the obtained supernatant using the Bradford method [42].

### 2.7. Determination of Antioxidant Enzyme Activities in the Heart Tissue

All enzyme activities were measured using a Shimadzu UV-160 spectrophotometer and a temperature-controlled cuvette holder.

#### 2.7.1. Determination of CAT Activity

Heart tissue CAT activity was determined spectrophotometrically according to the method of Beers and Sizer [43]. Absorbance change during hydrogen peroxide breakdown by CAT was directly tracked at 240 nm during one minute. One unit of enzyme activity (U) was defined as the decomposition of 1 *μ*mol hydrogen peroxide per minute under the test conditions.

#### 2.7.2. Determination of SOD Activity

The activity of SOD was determined spectrophotometrically at 480 nm by the epinephrine method [44]. This method is based on the capacity of SOD to inhibit autoxidation of epinephrine to adrenochrome at pH 10.5. One unit of SOD activity was defined as the concentration of the enzyme in the sample that caused 50% inhibition of the autoxidation of epinephrine at 26°C.

### 2.8. Determination of LDH Activity

Activity of LDH was determined by spectrophotometric measurement of the absorbance decrease at 340 nm during oxidation of NADH [45]. One unit of LDH activity (U) catalyzes the transformation of 1 *μ*mol of NADH per minute under the test conditions.

### 2.9. Determination of MDH Activity

Activity of MDH was determined by the method of Frieden and Fernandez [46]. Absorbance decrease was measured during oxidation of NADH at 340 nm. One unit of MDH activity (U) catalyzes the transformation of 1 *μ*mol of NADH per minute under the test conditions.

### 2.10. Determination of LDH Isoforms

Distribution of LDH isoforms was determined by the direct electrophoretic zymography according to Cunningham et al. [47]. Isoforms appeared on the gel as dark blue bands of formazan, which is formed after the reduction of nitroblue tetrazolium in the presence of the electron transfer mediator phenazinemetosulphate and NAD^+^ as a coenzyme. The activities of individual isoforms were semiquantitatively assessed as relative activity of total LDH activity and expressed in percentages.

### 2.11. Determination of MDH Isoforms

Distribution of MDH isoforms was determined by the direct electrophoretic zymography, according to the method of Yoshimura et al. [48]. Isoforms were visualized as dark blue bands of formazan, which is formed as a product of the reduction of nitroblue tetrazolium in the presence of phenazinemetosulphate as an electron transfer mediator and NAD^+^ as a coenzyme. The activities of individual isoforms were semiquantitatively assessed as relative activity of total MDH activity and expressed in percentages. LDH and MDH isoforms on obtained zymograms were analyzed using the ImageJ Q.42 software package.

### 2.12. Histological Analysis

Blocks of tissue were fixed by immersion in 4% neutral-buffered formaldehyde for at least 24 h [49]. The tissue was then dehydrated and embedded in paraplast. Tissue elements were cut with a microtome (Leica Reinhart Austria and Leica SM2000 R, Heidelberg, Germany). Sections of the heart tissue (5-*μ*m thick) were sampled from the analyzed specimens and stained with hematoxylin and eosin. This staining enables the morphological evaluation of changes in the heart, primarily the structure of the extracellular matrix, as well as the morphometric analysis and determination of the transversal diameter of the cardiomyocytes. Morphometric parameters were determined on the two cross section levels. The first represented the apex of the heart and the second the middle portion of the heart. For all the analyses, an Olympus BX41 microscope (Tokyo, Japan) with the Olympus C5060-ADU “wide zoom” camera was used (Tokyo, Japan). Measurements were performed using the image analyzer (ImageJ Q.42 software package).

### 2.13. Statistical Analysis

Data were expressed as mean ± SEM or median (minimal value and maximal value). Statistical comparison between the values obtained from the experimental groups was performed by one-way analysis of variance (ANOVA), followed by Tukey's post hoc test or by the Kruskal-Wallis test and Mann-Whitney *U* depending on the data distribution. SPSS 19.0 for Windows software package was used for statistical analyses. Differences were considered significant at *P* < 0.05.

## 3. Results

### 3.1. Body Weight and Heart Weight Index

All animals survived over the entire experimental period. Compared to the healthy rats, weight loss was observed in both the diabetic rat groups during the experimental period (*P* < 0.001, Table 1). In the DM group, the HWI was enhanced by 18.39% in comparison to that in the C1 group (*P* = 0.047, Table 1).

### 3.2. Biochemical Parameters

**Figure 1:**
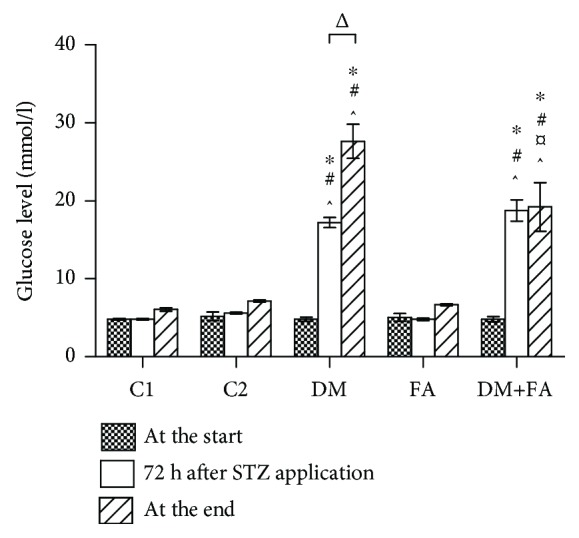
The glucose level (mmol/l) at the start of the experimental period, 72 h after STZ or saline administration, and at the end of the experimental period. ^∗^*P* < 0.05 versus the C1 group, ^#^*P* < 0.05 versus the C2 group, ^¤^*P* < 0.05 versus the DM group, ^^^*P* < 0.05 versus the FA group, and ^Δ^*P* < 0.05 comparing the glucose level 72 h after the STZ administration and at the end of the experimental period.

**Figure 2:**
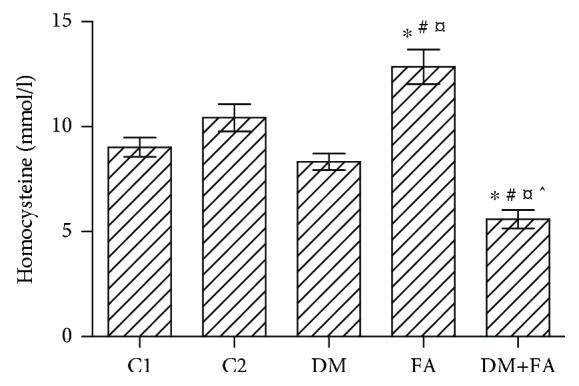
The serum homocysteine level (mmol/l) in the tested groups (*P* < 0.001). ^∗^*P* < 0.05 versus the C1 group, ^#^*P* < 0.05 versus the C2 group, ^¤^*P* < 0.05 versus the DM group, and ^^^*P* < 0.05 versus the FA group.

There was no statistical difference in the glucose level between the tested groups before the STZ treatment (*P* = 0.128). An elevated glucose level was found in all treated animals 72 h after the STZ administration (DM = 17.22 ± 0.646 mmol/l and DM+FA = 18.78 ± 1.35 mmol/l). At the end of the experimental period, the glucose level in the group DM+FA was still elevated in comparison to both the control groups, but significantly lower than that in the DM group (DM = 27.66 ± 2.185 mmol/l and DM+FA = 19.24 ± 3.122 mmol/l) (Figure 1). The serum insulin level and HOMA-IR were determined in the C1 group and the DM group to confirm the development of diabetes. In the DM group, the insulin level was significantly lower and HOMA-IR was significantly higher (*P* < 0.001) than those in the C1 group (Table 2). Statistically significant difference in the serum homocysteine level was found among the groups (*P* < 0.001, Figure 2). It was significantly increased in the FA group (12.86 ± 0.83 mmol/l) and decreased in the DM+FA group (5.59 ± 0.43 mmol/l). Serum cardiac tissue damage parameters revealed that there was no difference in LDH among the groups, while the troponin T level was significantly decreased in the DM group (Table 2). Haemostatic parameters exhibited significant difference among the groups. Fibrinogen was decreased in the DM group, while vWF was increased in both the diabetic groups (Table 2). Serum TC, HDL, and TG levels were significantly increased in the DM group in comparison to all other groups, while all these parameters were decreased in the DM+FA group in comparison to the DM group. However, the LDL level in the DM group was higher than in the C1 group and lower than in the other groups. The highest value of LDL was observed in the diabetic group with the folic acid treatment. AI was increased in the DM group, but even higher values were obtained in the C2, FA, and DM+FA groups in comparison to the C1 group (Table 3).

### 3.3. Antioxidant Enzyme Activities

**Figure 3:**
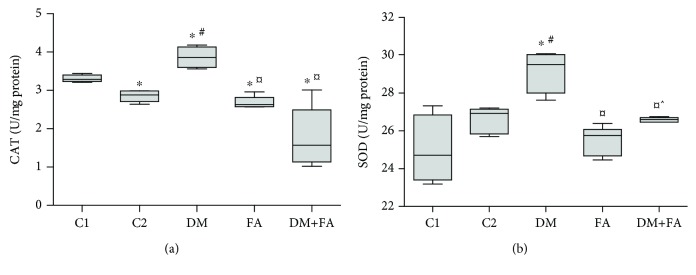
Antioxidant enzymes in the cardiac tissue: catalase (CAT) activity (a) and superoxide dismutase (SOD) activity (b). ^∗^*P* < 0.05 versus the C1 group, ^#^*P* < 0.05 versus the C2 group, ^¤^*P* < 0.05 versus the DM group, and ^^^*P* < 0.05 versus the FA group.

Antioxidant enzyme activities (CAT and SOD) in cardiac tissue homogenate were statistically different among the groups (*P* = 0.001 and *P* = 0.008, respectively). CAT activity was the highest in the DM group, while the administration of folic acid leads to decrease in its activity in both the healthy rats and the rats with induced DM (Figure 3(a)). Similarly, SOD activity has the highest values in the DM group and folic acid treatment significantly decreased its activity in rats with DM (Figure 3(b)).

### 3.4. LDH and MDH Activities

**Figure 4:**
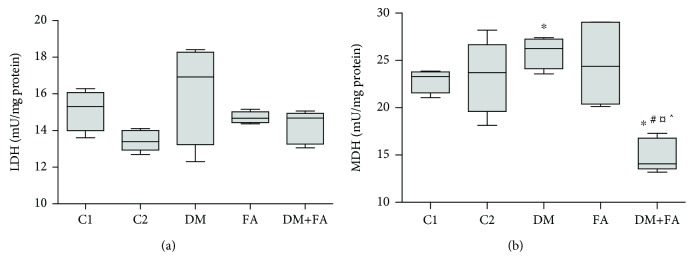
Lactate dehydrogenase (LDH) activity (a) and malate dehydrogenase (MDH) activity (b) in the cardiac tissue. ^∗^*P* < 0.05 versus the C1 group, ^#^*P* < 0.05 versus the C2 group, ^¤^*P* < 0.05 versus the DM group, and ^^^*P* < 0.05 versus the FA group.

There was no statistically significant difference in LDH activity among the groups (*P* = 0.074, Figure 4(a)), while MDH activity varies among the groups significantly (*P* = 0.013). It was increased in the DM group in comparison to the C1 group and decreased in the DM+FA group in comparison to all other groups (Figure 4(b)).

### 3.5. LDH and MDH Isoform Determination

**Figure 5:**
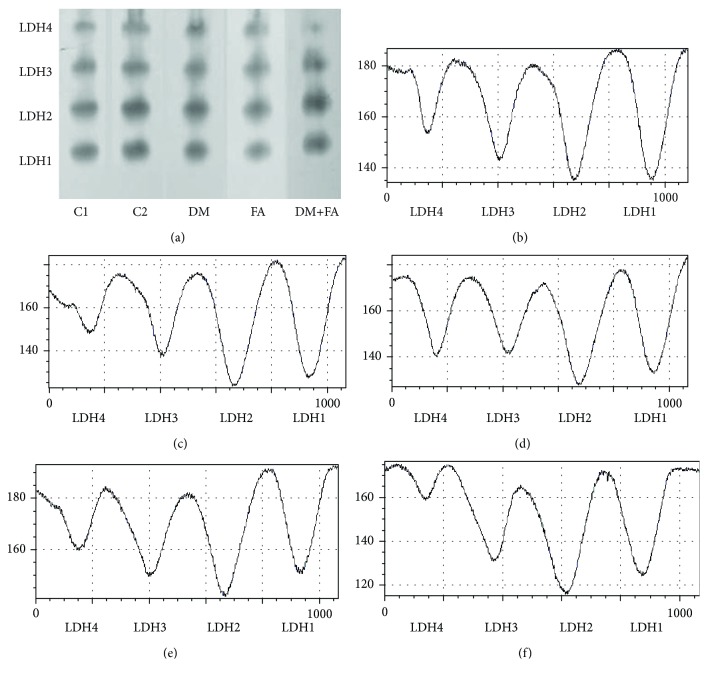
Relative activities of lactate dehydrogenase (LDH) isoforms in the cardiac tissue. Representative electrophoretic zymograms of LDH isoforms (a) and their densitometry in the C1 (b), C2 (c), DM (d), FA (e), and DM+FA (f) groups.

**Figure 6:**
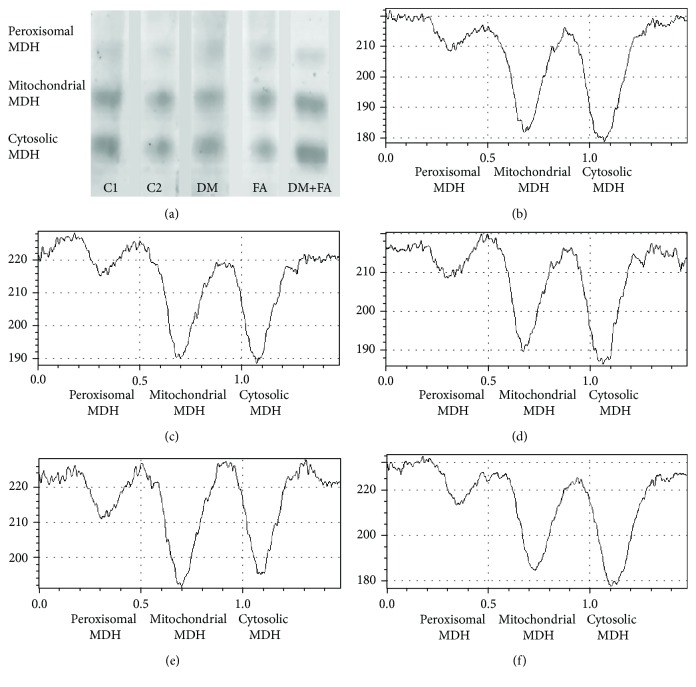
Relative activities of malate dehydrogenase (MDH) isoforms in the cardiac tissue. Representative electrophoretic zymograms of MDH isoforms (a) and their densitometry in the C1 (b), C2 (c), DM (d), FA (e), and DM+FA (f) groups.

**Figure 7:**
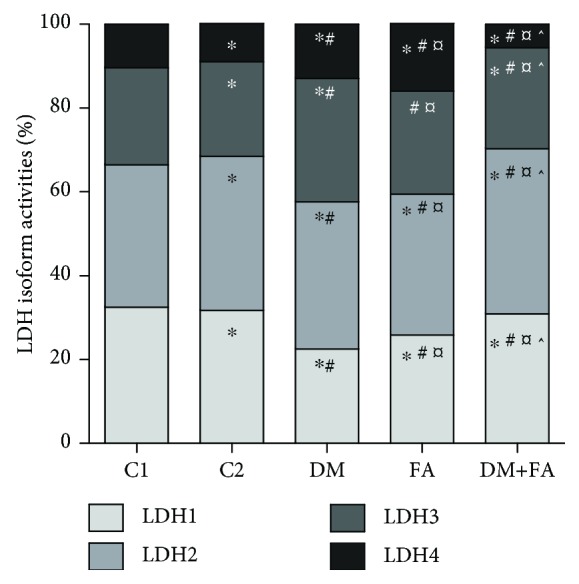
Relative activity of lactate dehydrogenase (LDH) isoforms in the cardiac tissue. ^∗^*P* < 0.05 versus the C1 group, ^#^*P* < 0.05 versus the C2 group, ^¤^*P* < 0.05 versus the DM group, and ^^^*P* < 0.05 versus the FA group.

**Figure 8:**
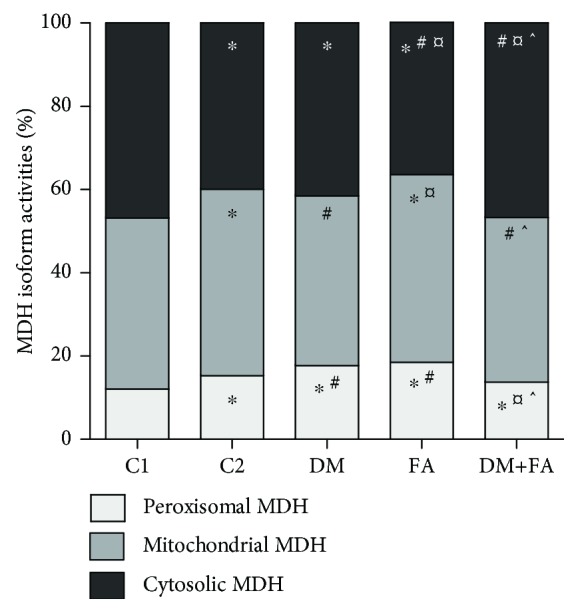
Relative activity of malate dehydrogenase (MDH) isoforms in the cardiac tissue. ^∗^*P* < 0.05 versus the C1 group, ^#^*P* < 0.05 versus the C2 group, ^¤^*P* < 0.05 versus the DM group, and ^^^*P* < 0.05 versus the FA group.

In the heart tissue of all tested animals, four LDH (LDH1, LDH2, LDH3, and LDH4) and three MDH (peroxisomal, mitochondrial, and cytosolic) isoforms were detected (Figures 5 and 6). In all groups, LDH2 isoform had the highest relative activity, then LDH1, LDH3, and LDH4 isoforms, but there were differences in the activities among the groups (*P* < 0.001, Figure 7). In the DM group, the decrease in LDH1 activity and the increase in activity of LDH3 and LDH4 in comparison to both the control groups are clearly noticed. Folic acid treatment of diabetic rats leads to the attenuation of these changes in activities and to a significant reduction of LDH4 relative activity. Also, relative activity of MDH isoforms was significantly different among the groups (*P* < 0.001, Figure 8). In the DM group, peroxisomal MDH activity was increased in comparison to that in the control groups, while in the DM+FA group, activity of this isoform was decreased.

### 3.6. Histological Analysis

**Figure 9:**
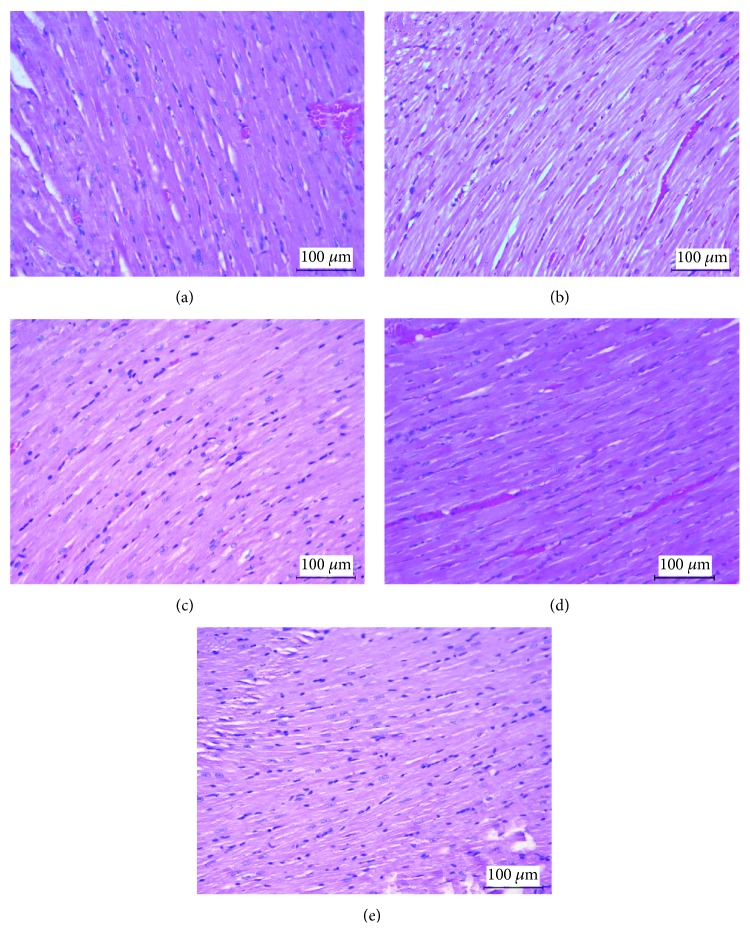
Representative histological slides of rats' hearts in the C1 (a), C2 (b), DM (c), FA (d), and DM+FA (e) groups at the middle heart cross section level (hematoxylin-eosin staining at magnification 200x). Left ventricular cardiomyocyte transversal diameters were significantly smaller in the DM, while the folic acid treatment of diabetic rats has reduced this decrease.

**Figure 10:**
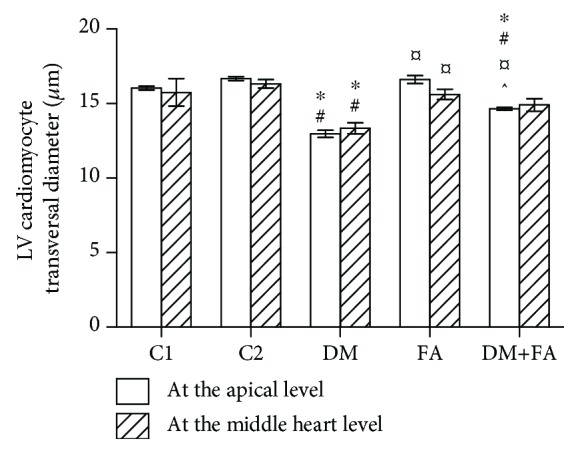
Left ventricular (LV) cardiomyocyte transversal diameter measured at the apical and middle heart levels. ^∗^*P* < 0.05 versus the C1 group, ^#^*P* < 0.05 versus the C2 group, ^¤^*P* < 0.05 versus the DM group, and ^^^*P* < 0.05 versus the FA group.

The myocardial fibres were arranged regularly, and the cardiomyocytes had physiological morphology in all tested groups (Figure 9). An increased heart surface area was obtained in the FA and DM+FA groups in comparison to the DM group (*P* = 0.007 and *P* = 0.015, respectively) at the apical level, while on the middle portion of the heart, an increased heart surface area was found only in the FA group in comparison to the C1 and DM groups (*P* = 0.006 and *P* = 0.012, respectively), but not with its correspondent control group (C2) and the DM+FA group. There were no differences in the left ventricular surface area among the groups, but the right ventricular surface area was increased in the FA group at the apical level. Both these surface areas were increased in the FA group at the middle portion of the heart level. Left ventricular wall thickness did not differ among the groups, at both tested levels, as well as right ventricular wall thickness at the middle portion of the heart level; however, it was decreased in the DM+FA group at the apical level (Table 4). There was no difference in left ventricular cardiomyocyte transversal diameters comparing three healthy groups, but these diameters were significantly smaller in the DM group at both tested levels (Figure 10). However, it was observed that folic acid treatment has reduced the decrease in the cardiomyocyte diameters.

## 4. Discussion

It is demonstrated that DM in a one-month period leads to different organ damages and increases oxidative stress [50], so the aim of this study was to evaluate the influence of folic acid administration during a four-week period on the oxidative stress parameters, as well as on LDH and MDH activities and their certain isoform distribution in the cardiac tissue of diabetic Wistar male rats.

Administration of STZ is one of the ways to induce DM type I in rodents [51]. In order to confirm that STZ (100 mg/kg) induced DM, the insulin level was measured and the HOMA-IR index was calculated in the C1 and DM groups. Induction of DM and increased insulin resistance in the tested animals was confirmed by an increased glucose level and HOMA-IR, as well as by a decreased insulin level in the DM group. In our experiments, all the animals treated with STZ had an elevated glucose level 72 h after the STZ administration. As it is expected in DM type I, STZ-treated rats had also lower body weight than controls [52, 53]. Application of folic acid attenuated hyperglycemia (however, glucose level values remained still higher than 11.0 mmol/l), while body weight of diabetic rats treated with folic acid did not change in comparison to diabetic rats without the treatment. Oxidative stress and inflammation that exist in diabetes promote cardiac hypertrophy and individually cardiomyocyte hypertrophy [54, 55]. A cardiac hypertrophy is also one of the signs of a diabetic cardiomyopathy [56, 57]. In this work, cardiac hypertrophy was confirmed in diabetic rats by a higher HWI. An increased HWI in the DM group indicated that a decrease in the heart weight did not follow a decrease in the body weight that was substantially reduced. Other authors have also determined cardiac hypertrophy by evaluation of the HWI and obtained similar results [40, 54]. Although we showed a cardiac hypertrophy that indicates a diabetic cardiomyopathy, the enzyme indicators of cardiomyocyte damage had not been increased in the serum. There were no differences in the serum LDH values, while the troponin T was even lowered in the DM group. Increased serum LDH concentration occurs in cardiomyocyte damage [58]. In this study, the serum LDH level did not differ between the groups, indicating that there was no cardiomyocyte damage; also, histological analysis did not reveal signs of necrosis. On contrary, other authors obtained increased serum LDH [40], as well as serum troponin I [54], but their research periods lasted longer (8 weeks). This may indicate that the absence of the alteration of the serum LDH and decreased serum troponin T are due to the short time of exposure to diabetes. All serum lipid profile parameters were elevated in the DM group. Folic acid treatment of diabetic rats increased LDL and decreased both the HDL and TG levels. Our results reveal that folic acid elevates the homocysteine level in the healthy rats and decreases its level in the diabetic group, which is also in accordance with other authors' results [21]. Changes in the HOMA-IR and biochemical markers indicate the existence of metabolic syndrome in addition to DM in the DM group.

Since increased oxidative stress and changes in antioxidant capacity are considered as an important mechanism in the pathogenesis of DM and its complications [14], oxidative stress examination in DM is of highly importance. Intensity of ROS generation modulates the response to oxidative stress. It is demonstrated that their low concentrations stimulate antioxidant defence and increase antioxidant enzyme activity, while high concentrations inhibit enzymatic activity that will lead to further cellular damage [59]. In this research, CAT and SOD activities were increased in the cardiac tissue of diabetic rats. Similar results were obtained by other authors [50, 60]. However, some researchers have demonstrated decreased CAT and SOD activities [15, 40, 54]. Our experimental period lasted four weeks, while in most of the studies that demonstrated decreased activities of antioxidant enzymes, the experimental period was longer. The increased values of CAT and SOD activities obtained by Strother et al. [50] are in concordance with our results, and their experimental period was of a similar duration as ours (thirty days). Since activities of antioxidant enzymes depend on ROS concentration, the possible cause of our results is that during the period of four weeks, ROS production due to DM was just slightly increased and that had stimulated the increase in CAT and SOD activities. In this research, folic acid treatment had a beneficial effect on diabetic rats since it decreased the value of both enzyme activities. As folic acid has an antioxidant effects, it is expected that its administration decreases the production of ROS and consequently reduce the activities of these enzymes. It is demonstrated that folic acid can reduce the production of superoxide radicals that is catalyzed by nitric oxide synthase (NOS) [61]. This enzyme needs the presence of the cofactor tetrahydrobiopterin (BH_4_) and substrate L-arginine for optimal function and NO production. When there is limited availability of the cofactor or substrate or when oxidative stress is elevated, NOS can destabilize and uncouple and it starts producing superoxide radicals instead of NO [62]. It is supposed that positive folic acid effects are mediated by its primary circulating metabolite 5-methyl tetrahydrofolate (5-MTHF). 5-MTHF increases the bioavailability of the BH_4_, interacts with NOS directly, and scavenges reactive oxygen species, specifically superoxide radicals, and indirectly by increasing BH_4_ availability, 5-MTHF stimulates NO synthesis and prevents superoxide production [61]. Based on the previous study, we can conclude that the decreased activities of SOD and CAT in the cardiac tissue of diabetic rats with the folic acid treatment could be the consequence of the reduced production of superoxide radical due to the effect of folic acid. Thus, strategies that reduce ROS production may have effects in recovering myocardial function in DM.

There was no difference in total LDH activity in cardiac tissue homogenate among the groups. By comparing LDH isoforms, we have revealed that in the DM group relative activity of LDH1 was decreased, while relative activity of LDH4 was increased. Decreased activities of LDH1 and LDH2 might indicate reduced formation of pyruvate [7] and insufficient supply in oxygen. Also, an increase in LDH4 activity may indicate that there is hypoxic condition in the body [7]. According to the previous study, our results probably mean that there was insufficient oxygen supply and therefore shift to anaerobic metabolism in diabetic rats' hearts. Treatment of diabetic rats with folic acid increased LDH1 relative activity almost to the control level and decreased LDH4 activity to the level lower than in the control groups. Total MDH activity was the highest in the DM group, while the folic acid treatment decreased significantly its activity. The increase in MDH activity is expected during gluconeogenesis [63] that occurs in the diabetic state [64]. There are no published papers about folic acid influence on the total cardiac MDH activity. Increased activity of peroxisomal MDH was obtained in the DM group, while mitochondrial and cytosolic isoform of MDH had lower activities. Mitochondrial MDH has a significant role in the citric acid cycle [10]. The enzymes of the citric acid cycle are involved in the maintenance of the reduced redox state in mitochondria with the aim of generating adenosine triphosphate via oxidative phosphorylation [65]. Decreased mitochondrial MDH activity in diabetic rats that indicates mitochondrial damage due to DM was confirmed in different studies [66–68]. It is demonstrated that hyperglycemia increases oxidative stress and endothelial cell apoptosis through ROS overproduction at the mitochondrial transport chain level [69] and that the ROS are involved in mitochondrial damage and in the citric acid cycle enzyme activity decrease [70]. Therefore, increased ROS production in hyperglycemic rats may lead to decreased TCA cycle enzyme activities [67]. Other authors have demonstrated changes in different peroxisomal enzyme activities due to DM, but they did not test the MDH activity [71]. The effect of diabetes on peroxisomes may be due to the increased supply of fatty acids and due to increased oxidative stress [71], which is in accordance with our results.

Although we have demonstrated cardiac hypertrophy in the DM group by HWI calculation, on histological measurements, there were no differences in the left ventricular surface area and left ventricular wall thickness. It has been found that folic acid treatment causes an increase in the area of the left and right ventricles in healthy rats. Our results showed decreased left ventricular cardiomyocyte transversal diameters. Similar results were obtained by other researchers who, in addition to the reduced cardiomyocyte diameter, showed that there was a decrease in the thickness of the left ventricular wall in diabetic rats [72]. In contrast to our results, Rabadiya et al. obtained increased left ventricular wall thickness and left ventricular cardiomyocyte diameter [73], while other authors demonstrated that there was no difference in left ventricular cardiomyocyte diameter [15].

## 5. Conclusions

Diabetes mellitus still represents a global public health problem with an increasing incidence and prevalence [74] that is characterized by multiple organ damages and complications. Therefore, in addition to conventional medical treatment, the use of supplements is of great importance, in order to prevent or reduce the DM complications. Obtained results demonstrated that DM type I has led to numerous changes in the cardiac tissue enzyme activities and in the cardiomyocyte diameters, as well as in cardiovascular biomarkers during the period of only four weeks, but also demonstrated that there are positive effects of the folic acid treatment in diabetic rats. In milieu of oxidative stress caused by experimentally induced DM, the application of folic acid leads to a cellular response characterized by antioxidative enzymes and MDH activity decrease. Our results demonstrated that the treatment with folic acid significantly reduced the blood glucose level and alleviated the changes of other tested parameters. We would like to highlight that the administration of folic acid in rats with experimentally induced diabetes has beneficial and protective effects. The folic acid treatment seems to have the promising results in diabetic rats. However, further studies are required, to establish causality between antioxidant supplementation and cellular response regarding the examined parameters.

## Figures and Tables

**Table 1:** Body weight at the start and at the end of the experimental period and heart weight index of experimental animals.

Parameters	Groups (mean ± SEM)					*P* value
	C1	C2	DM	FA	DM+FA	
Body weight (g) at the start	145.6 ± 2.8	146.6 ± 1.9	145.4 ± 2.4	145.4 ± 2.4	151.3 ± 3.4	0.286
Body weight (g) at the end	253.1 ± 7.9	364.0 ± 8.3^∗^	181.0 ± 7.8^∗^^,#^	363.8 ± 7.8^∗^,¤	192.3 ± 9.3^∗^^,#,^^	<0.001
Heart weight index (mg/g)	2.99 ± 0.14	3.02 ± 0.08	3.54 ± 0.05^∗^	3.39 ± 0.17	3.46 ± 0.12	0.008

^∗^ *P* < 0.05 versus the C1 group, ^#^*P* < 0.05 versus the C2 group, ^¤^*P* < 0.05 versus the DM group, and ^^^*P* < 0.05 versus the FA group.

**Table 2:** Biochemical parameters in serum and plasma of the experimental animals.

Parameters	Groups (mean ± SEM)					*P* value
	C1	C2	DM	FA	DM+FA	
Insulin (mU/l)	26.8 ± 1.0	/	20.2 ± 0.9^∗^	/	/	<0.001
HOMA-IR	7.2 ± 0.4	/	26.6 ± 2.2^∗^	/	/	<0.001
LDH (U/l)	3887.1 ± 358.5	4438.4 ± 331.7	3913.7 ± 249.3	3490.5 ± 429.2	4212.5 ± 357.1	0.378
Troponin T (ng/l)	25.8 ± 6.2	23.1 ± 2.4	6.4 ± 1.0^∗^^,#^	34.7 ± 0.0^¤^	27.6 ± 8.0^¤^	0.003
Fibrinogen (g/l)	2.6 ± 0.3	2.2 ± 0.1	1.2 ± 0.2^∗^^,#^	2.2 ± 0.1^¤^	2.1 ± 0.1^¤^	0.004
von Willebrand factor (%d.N.)	214.3 ± 30.4	99.4 ± 4.3^∗^	467.9 ± 43.6^∗^^,#^	46.1 ± 16.3^∗^^,#,¤^	379.5 ± 34.9^∗^^,#,^^	<0.001

HOMA-IR: homeostasis model assessment of insulin resistance; LDH: lactate dehydrogenase. ^∗^*P* < 0.05 versus the C1 group, ^#^*P* < 0.05 versus the C2 group, ^¤^*P* < 0.05 versus the DM group, and ^^^*P* < 0.05 versus the FA group.

**Table 3:** Lipid profile parameters in serum of the experimental animals.

Parameters	Groups (mean ± SEM)					*P* value
	C1	C2	DM	FA	DM+FA	
TC (mmol/l)	1.38 ± 0.06	1.53 ± 0.07^∗^	2.32 ± 0.13^∗^^,#^	1.52 ± 0.07^¤^	1.48 ± 0.13^¤^	<0.001
HDL (mmol/l)	1.12 ± 0.05	0.64 ± 0.03^∗^	1.51 ± 0.11^∗^^,#^	0.60 ± 0.02^∗^^,¤^	0.49 ± 0.09^∗^^,¤^	<0.001
LDL (mmol/l)	0.09 ± 0.01	0.53 ± 0.06^∗^	0.28 ± 0.06^∗^^,#^	0.53 ± 0.06^∗^^,¤^	0.66 ± 0.08^∗^^,¤^	<0.001
TG (mmol/l)	0.58 ± 0.05	0.78 ± 0.05^∗^	2.03 ± 0.22^∗^^,#^	0.85 ± 0.1^∗^^,¤^	0.59 ± 0.18^#,¤,^^	<0.001
AI	0.078 ± 0.01	0.812 ± 0.07^∗^	0.189 ± 0.05^∗^^,#^	0.875 ± 0.09^∗^^,¤^	0.900 ± 0.19^∗^^,¤^	<0.001

TC: total cholesterol; HDL: high-density lipoprotein; LDL: low-density lipoprotein; TG: triglycerides; AI: atherogenic index. ^∗^*P* < 0.05 versus the C1 group, ^#^*P* < 0.05 versus the C2 group, ^¤^*P* < 0.05 versus the DM group, and ^^^*P* < 0.05 versus the FA group.

**Table 4:** Cardiac histomorphometry parameters measured at two cross section levels (apical and middle heart).

Parameters		Groups (mean ± SEM)					*P* value
	Cross section level	C1	C2	DM	FA	DM+FA	
LV wall thickness (mm)	Apical	2.2 ± 0.17	1.8 ± 0.14	1.8 ± 0.13	2.1 ± 0.08	2.0 ± 0.11	0.109
	Middle	2.6 ± 0.2	2.4 ± 0.1	2.3 ± 0.0	2.6 ± 0.2	2.1 ± 0.1	0.098
RV wall thickness (mm)	Apical	0.7 ± 0.03	0.6 ± 0.03	0.6 ± 0.05	0.6 ± 0.02	0.5 ± 0.01^∗^^,#,^^	0.002
	Middle	0.7 ± 0.07	0.7 ± 0.03	0.7 ± 0.02	0.7 ± 0.03	0.6 ± 0.03	0.414

LV: left ventricle; RV: right ventricle. ^∗^*P* < 0.05 versus the C1 group, ^#^*P* < 0.05 versus the C2 group, and ^^^*P* < 0.05 versus the FA group.

## Data Availability

The data used to support the findings of this study are available from the corresponding author upon request.

## Abbreviations

AI: Atherogenic index
CAT: Catalase
DM: Diabetes mellitus
HDL: High-density lipoprotein
HOMA-IR: Homeostasis model assessment of insulin resistance
HWI: Heart weight index
LDH: Lactate dehydrogenase
LDL: Low-density lipoprotein
LV: Left ventricle
MDH: Malate dehydrogenase
PMSF: Phenylmethylsulfonyl fluoride
ROS: Reactive oxygen species
RV: Right ventricle
SOD: Superoxide dismutase
STZ: Streptozotocin
TC: Total cholesterol
TG: Triglycerides
vWF: von Willebrand factor
NOS: Nitric oxide synthase
5-MTHF: 5-Methyl tetrahydrofolate
BH_4_: Tetrahydrobiopterin.
